# Letter in response to the letter to the editor of archives of toxicology by Woegerbauer et al. (2016)

**DOI:** 10.1007/s00204-016-1860-2

**Published:** 2016-10-05

**Authors:** Ralf Wilhelm, Christian Kohl, Joachim Schiemann, Pablo Steinberg

**Affiliations:** 1 Institute for Biosafety in Plant Biotechnology, Julius Kühn-Institut, Erwin-Baur-Str. 27, Quedlinburg, 06484 Germany; 2 Institute for Food Toxicology and Analytical Chemistry, University of Veterinary Medicine Hannover, Foundation, Hannover, 30173 Germany

We thank our Austrian colleagues for entering into an open discussion on the contentious topic regarding the necessity of animal feeding trials with whole genetically modified (GM) food/feed based on the outcome of the EU-funded project GRACE. We agree that an evidence-based and thorough scientific discussion should precede any far-going decision on mandatory trials to be used in the risk assessment of whole GM food/feed to prevent undermining its scientific soundness.

Woegerbauer et al. (2016) refer to the brochure “Conclusions and Recommendations” (GRACE 2015a), which was published on the website of the project, and, specifically, to “PART I: Conclusions and recommendations on animal feeding studies and alternative approaches with regard to Article 12, Implementing Regulation (EU) No. 503/2013” therein.

Hereby we answer to the different points raised by Woegerbauer et al. (2016) as follows:

(1): “…it is astonishing that in this final report the GRACE consortium appears to have refrained from communicating uncertainties…”

*Answer* To clarify this issue on which Woegerbauer et al. (2016) have placed special emphasis, it should be noted that the brochure “Conclusions and Recommendations” summarizes the experiences and opinions of the GRACE project consortium by listing central statements and highlights key issues for discussion from the point of view of the consortium. The brochure “Conclusions and Recommendations” is neither a scientific report nor a comprehensive assessment paper and was never claimed to be such a document. At this stage, it is important to recall that one of the central objectives of the GRACE project was to conduct animal feeding trials and in vitro studies to compare the added value of 90-day feeding trials of whole GM food/feed with that of advanced state-of-the-art analytical, in vitro and in silico tools by making use of the genetically modified MON810 maize as test material. In no case was the GRACE project consortium requested to perform a full risk assessment on the GM maize MON810, and none of the scientific documents published by the GRACE project consortium as well as the brochure “Conclusions and Recommendations” were considered to replace a full risk assessment of the GM maize MON810. The mandatory character of the 90-day animal feeding trial with whole GM food/feed in the Implementing Regulation (EU) No. 503/2013 assumes that its performance generally and per se reduces the level of uncertainty. Our trials with the GM maize MON810 have shown that this untargeted approach does not achieve an added value. In the context of the “Guidance on Uncertainty in EFSA Scientific Assessment” (EFSA Scientific Committee 2016), it is unlikely that such untargeted feeding trials can generally and substantially support the uncertainty analyses of an assessment question as well as the decision making.

Regarding the issue of uncertainty analyses raised by our Austrian colleagues, we interpret the paper by the EFSA Scientific Committee (2016) as a guideline targeting the handling of (risk) assessment questions rather than single studies. Nevertheless, EFSA refers to uncertainty analyses in its guidance document published in 2011 (EFSA 2011): “A specific chapter on assumptions and uncertainty analysis should be included in the study report. Any uncertainties (in addition to natural variation in biological endpoints) in the design of the experimental model which might influence the power of the experiment should be highlighted and quantified as far as possible. The assumptions underlying the statistical analysis should be reported and tested for robustness.” We are of the opinion that the complete data sets as well as the detailed description of the trials and the obtained results provided with the scientific publications, each of them including a detailed statistical report with an explanation regarding the statistical models used, sufficiently display the trial-specific uncertainties.

In addition, the final report on the stakeholder consultations “Assessing Feeding Studies and Alternative Approaches for GMO Risk Assessment” (GRACE 2015b) is available at the GRACE project website (www.grace-fp7.eu) and lists in detail questions and answers regarding the performances of the feeding trials. This comprehensive pool of information is provided to help assessors, decision and policy makers to evaluate the added value of animal feeding trials with whole GM food/feed, and the “Conclusions and Recommendations” brochure depicts the interpretation of the findings by the GRACE project consortium.

(2): “…we miss a clearly structured description of the ‘strengths and limitations’ of 90-day feeding trials in rodents and alternative studies as announced in the introductory section of the final ‘Conclusions and Recommendations’ (GRACE Consortium 2015).”

*Answer* The GRACE project consortium stated that GRACE aims “to provide recommendations on the appropriateness of these tools for the risk assessment of GM crops by considering the scientific strengths and limitations of the different approaches” (GRACE 2015a). During the elaboration of the brochure “Conclusions and Recommendations,” the scientific strengths and limitations of the different approaches were thoroughly considered and discussed at a workshop held on the 7th and 8th of October 2015 in Vienna (see point 3 and GRACE 2015b). Due to the focus of the brochure “Conclusions and Recommendations,” as described in the answer to point 1, not all strengths and limitations accompanying the respective approaches have been listed.

(3): “Although stakeholder involvement is emphasized on several occasions in the booklet, GRACE refrains from reporting any discrepancies which had arisen during extended oral and written discussions on the project design and on the interpretation of the results.”

*Answer* Stakeholders were actively involved in the discussions on the project design, the interpretation of the results as well as the elaboration of the project conclusions and recommendations. This active stakeholder involvement is documented by the following consultation reports, all of them having been published (i.e., being publicly available):

- GRACE Stakeholder Consultation on animal feeding studies and in vitro studies in GMO risk assessment, March 2013 (GRACE 2013a).
- Responses of GRACE team members to questions and comments raised by stakeholders, December 2013. Addendum to the Stakeholder Consultation Report published in March 2013 (GRACE 2013b).
- GRACE Stakeholder Consultation on animal feeding studies in GMO risk assessment: Chronic toxicity (1-year) study and subchronic toxicity and longitudinal metabolomics study and responses of GRACE team members to questions and comments raised by stakeholders, July 2014 (GRACE 2014).
- Draft results of the 90-day animal feeding studies and progress of the in vitro, in silico and analytical studies with GM maize, June 2015 (GRACE 2015c).
  Raw data were provided to those participants that signed a non-disclosure agreement.
- Stakeholder Consultation on the results of the GRACE feeding studies as well as of the in vitro, in silico and analytical studies and on general conclusions and recommendations, November 2015 (GRACE 2015b).
  Raw data were provided to those participants that signed a non-disclosure agreement.

Finally, draft conclusions and recommendations were provided by the GRACE project consortium for discussion during a workshop held on the 7th and 8th of October 2015 in Vienna (GRACE 2015a). All stakeholder responses, either provided during the workshop or submitted in writing, were considered during their finalization. GRACE responses to the comments are thoroughly documented in the corresponding consultation report (GRACE 2015b).

(4): “Limitations and weaknesses of the overall study approach (e.g. testing of only a single active principle; no empirical analysis of stacked events or GMOs with complex alterations of metabolic pathways or events coding for different modes of action etc.) are not clearly communicated.”

*Answer* The Implementing Regulation (EU) No. 503/2013 does not suggest a hierarchical and targeted assessment process prior to the performance of 90-day feeding trials with whole GM food/feed. It generally assumes that the approach reduces uncertainty and increases the confidence of the consumers. The feeding trials performed by GRACE demonstrated that this assumption does not generally hold true and this is in particular the case if there is no previous indication of a potential adverse effect by other approaches. So far, it has not been shown that rodent feeding trials are a sensitive test system to identify adverse effects induced by whole GM food/feed and that they are more sensitive than other available methods. The limitations of the approach have not only been demonstrated by the GRACE project but also, e.g., by analyzing GM potatoes with an altered glycoalkaloid spectrum (Langkilde et al. 2012). Therefore, the GRACE team suggested to follow an overall approach that is based on the identification of triggering findings (e.g., by a detailed analysis of the plant material composition), which in turn may justify an animal feeding trial with whole GM food/feed. A priori and without a case-specific consideration—e.g., with regard to a complex alteration of metabolic pathways—rodent feeding trials with whole GM food/feed do not provide an added scientific value for the risk assessment of GM crops.

(5): “The ‘Conclusions and Recommendations’ imply general validity for the toxicological assessment of plant-derived GMOs but the project design was not intended to provide experimental evidence in support of these generalizations. ‘GRACE is expected to provide sound conclusions and recommendations on the adequacy of the approaches tested in the frame of GRACE’ … and … is in the position to report results and conclusions on experience gained with MON810. But GRACE extrapolates far beyond this scope. This constitutes a bias which should be clearly communicated to the risk managers in the respective booklet as this is the basis for informed decision making.”

*Answer* See answer to point 4.

(6): “GRACE highlights the importance of a ‘targeted and testable hypothesis’ as trigger for animal experiments but does not communicate that unintended effects may be - in certain cases - unpredictable and unexpected and, thus, might not be detectable by a hypothesis-driven approach.”

*Answer* The detection of unpredictable effects should indeed be based on screening methods of considerable sensitivity. Rodent feeding trials with whole GM food/feed performed according to the OECD Test Guidelines, in contrast to those with chemicals, have failed to demonstrate this degree of sensitivity (see, e.g., Langkilde et al. 2012).

(7): “In our opinion the selection of certain project partners was not as optimal as would have been required by the demanding specific conditions related to toxicity testing of GMOs in whole food and feed. In particular the animal experimentation indicates some flaws which might have been relevant for the explanatory power of the results as a whole: The observation of circadian effects during trial A and trial B … might be indicative for a non-optimal execution of these experiments, as this kind of effect would not have emerged if the studies were performed exactly according to OECD TG 408, EFSA 2011, and GLP requirements (EFSA 2011; OECD 1998).”

*Answer* The European Commission required in the project formulation and negotiation phases that the feeding trials should be performed in an institution not being involved before in the commercial testing of whole GM food/feed to avoid any kind of conflicting interests. It has to be pointed out that the Laboratory of Toxicology of the Slovak Medical University has a long-lasting experience in the performance of 90-day subchronic toxicity studies with pesticides and was chosen by the GRACE project consortium because of this expertise. In the frame of GRACE, the Slovakian colleagues successfully performed four 90-day feeding trials and a 1-year feeding study, and in no case were there any indications that they were not able to cope with the feeding trials as well as the subsequently performed blood and urine analyses. It is important to note that none of the parameters measured according to the OECD TG 408 in the 90-day feeding trials A and B were affected by the circadian rhythm of the animals. The influence of the circadian rhythm on the omics data underlined the need for a targeted adjustment of the study design when—in addition to a routine toxicological feeding trial according to the OECD TGs—additional methods that track physiological dynamics are applied. Hence, the statement by Woegerbauer et al. (2016) that “this kind of effect would not have emerged if the studies were performed exactly according to OECD TG 408, EFSA 2011, and GLP requirements” is misleading in more than one way as, e.g., a randomized sampling during necropsy is not suggested by the corresponding documents. The 90-day feeding trials were performed under GLP conditions by taking into account the OECD TG 408 as well as the EFSA recommendations (those parts that were not performed under GLP conditions are explicitly mentioned in the corresponding study plan). We are of the opinion that discrediting the Slovakian colleagues that performed the animal feeding studies in the frame of the GRACE project on a high technical and scientific level conflicts with a fair scientific debate.

(8): “The necessity to include conventional control maize varieties (2 of them contaminated with MON810) as surrogates for missing historical control data is another indication for a non-optimal expertise on this type of whole food/feed studies in rodents.”

*Answer* The inclusion of conventional maize varieties as surrogates for missing historical control data is explicitly recommended by EFSA (EFSA Scientific Committee 2011) and is in no way related to the expertise of an animal housing facility to perform 90-day subchronic toxicity studies. A statement regarding the expertise of the Laboratory of Toxicology of the Slovak Medical University, which performed four 90-day feeding trials and a 1-year feeding study with diets containing the GM maize MON810 in the frame of the GRACE project, is included under point 7.

(9): “A trend analysis considering trends and patterns in statistically non-significant differences (taking into account all applied diets, but not disregarding the primary importance of the comparison of GM and non-GM near-isogenic control groups) is missing although this approach would provide valuable information.”

*Answer* The results of the descriptive statistical analyses revealed no trends that would have justified a further analysis. Any interested party can view these analyses in form of statistical reports for the already published 90-day feeding trials A and B and the 1-year feeding trial with the GM maize MON810 on the website www.cadima.info. Moreover, the freely available data sets enable any third party to run their own analyses.

(10): “In support of its conclusions GRACE refers on several occasions to the CADIMA database and to (envisioned) scientific publications (GRACE Consortium 2015). At the time of writing the CADIMA database was not fully functional and contained only the data from trial A and B… However, it is questionable whether a database lacking a substantial part of the project results and unpublished papers are the appropriate basis for risk managers to decide in a timely manner on possible amendments of Commission Implementing Regulation 503/2013 …”

*Answer* The GRACE project consortium decided from the beginning to publish the results of the feeding trials in form of papers in an internationally recognized scientific journal and to provide open access to the detailed feeding trial data in the CADIMA database after acceptance of the manuscripts. In this context, it is important to note that, as already indicated under point 3, stakeholders had access to the raw data of the feeding trials before the corresponding publication of the manuscripts if they signed a non-disclosure agreement. The peer review of the very detailed manuscripts turned out to be considerably time-consuming, but in the meantime the outcome of the first two 90-day feeding trials A and B (Zeljenková et al. 2014), the outcome of the 1-year feeding trial (Zeljenková et al. 2016) and the description of the statistical methods used to analyze the data obtained in the feeding trials (Schmidt et al. 2016a) have been published in *Archives of Toxicology*. As promised, now that the papers have been published, the corresponding data sets can be downloaded on the website https://www.cadima.info/index.php/area/publicAnimalFeedingTrials. Hence, the risk assessors do have all the data they need to decide on the future of the Implementing Regulation (EU) 503/2013. A manuscript reflecting the results of all 90-day feeding trials conducted in GRACE and including the description of the last two 90-day feeding trials performed (the so-called feeding trials D and E) very recently has been accepted for publication in *Archives of Toxicology* (Schmidt et al. 2016b).

(11): “GRACE applied two conventional control maize varieties which were contaminated with MON810 in fact constituting GM diets with a GM content of approx. 1 % (Zeljenková et al. 2014). According to the European GMO legislation currently in force these diets would have had to be labelled as being genetically modified (European Commission 2003). So it is at least questionable whether these contaminated varieties are eligible to establish historical control data which should be mandatorily generated by non-GM lines (EFSA 2011).”

*Answer* First of all, the decision to label plants and products thereof containing more than 0.9 % of a GM organism (European Commission 2003) as being genetically modified is a political one and in no way indicates adverse effects. Moreover, Woegerbauer et al. (2016) mention that the diet containing the conventional 1 maize variety (PR33W82) used in the 90-day feeding trials A and B was contaminated with GM MON810 at a level of approx. 1 %, but they do not mention that: (1) GM maize MON810 was not detected in the diet containing the conventional 2 maize variety (SY-NEPAL); (2) the 13 hematology and 15 clinical biochemistry parameters measured in the blood of rats being fed the control, conventional 1 and conventional 2 diets were, with single exceptions, comparable. Taken together, even at a contamination level of 1 % MON810 the rat groups being fed conventional maize-containing diets are eligible to establish historical control data.

Woegerbauer et al. (2016) allude to an electronic annex to their Letter to the Editor, in which a number of further concerns are listed and which has been published on the GRACE homepage. The GRACE consortium will answer to all the issues raised in the above-mentioned electronic annex in due time.
